# Allied eye health professionals in eye care services in Nepal

**Published:** 2020-12-31

**Authors:** Rajendra Gyawali, Rabindra Adhikary, Himal Kandel

**Affiliations:** 1 President: Better Vision Foundation, Kathmandu, Nepal.; 2 Master of Optometry Student: Tilganga Institute of Ophthalmology, Kathmandu, Nepal.; 3 Kornhauser (postdoctoral) Research Associate: Save Sight Institute, Sydney Medical School, Sydney.

**Despite challenges, Nepal's eye care system is witnessing significant improvement, including an increase in eye hospitals and better cataract surgical coverage.**

**Figure:**
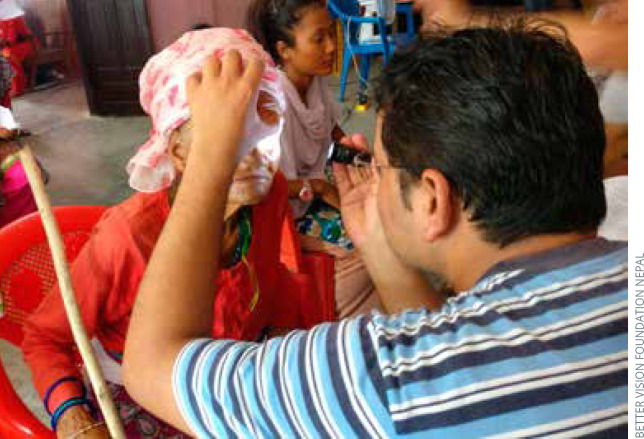
Allied eye care professionals are engaged in the outreach eye camps in remote communities in Nepal. **NEPAL**

In the last few decades, Nepal's eye care system has made remarkable progress in reducing the magnitude of blindness. Some of the achievements include:

- A decline in the prevalence of blindness from 0.84 per cent (1980) to 0.35 per cent (2019),^1^
- Increased cataract surgical coverage (for people with visual acuity less than 3/60) from 35 per cent (1980) to 85 per cent (2011),^2^ and
- The elimination of trachoma as a public health problem in 2019.^3^

From just one eye hospital in 1980, Nepal today has more than 40 secondary and tertiary hospitals, ophthalmic departments and more than 100 district and community eye care centres. The last three decades also witnessed significant progress in the development of the eye care workforce, making the country self-reliant in most of the human resources for its eye care services.^3^ Allied eye health professionals have played a major role in these achievements.

The WHO Global Action Plan 2014-19 recognises a range of health care professionals as allied ophthalmic personnel.^4^ Ophthalmic assistants/technicians, ophthalmic nurses, opticians, and ophthalmic photographer/imagers are the major allied health personnel in Nepal's eye care system. Nepal also has optometry technicians, orthoptists, vision therapists, ocularists and dedicated ophthalmic administrators, but in limited numbers.

**Figure 2:**
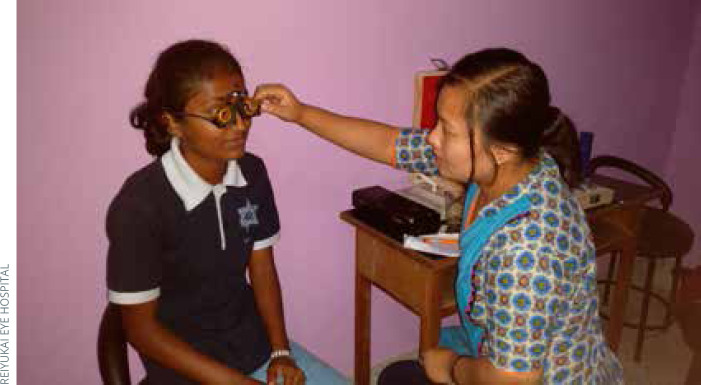
An ophthalmic Assistant performs refraction in a school student in a community eye centre.

## Ophthalmic assistants

Ophthalmic assistants (OAs) form the backbone of the rural eye care structure in Nepal, where the services of ophthalmologists and optometrists are not sufficient to meet the need. Since 1981, over 1,000 OAs have been trained to assist ophthalmologists in outpatient departments, operating theatres and community outreach camps.^3^ Their training included identification and management of common eye conditions and refractive errors. They also work as facility managers in the district and community eye centres. They are usually situated within the district headquarters, or community eye centres especially in remote, mountainous regions.

## Opticians

It is estimated that about 350 formally trained opticians and an equal number of unregistered, informally trained dispensers are providing spectacle dispensing services in various outlets, mainly in urban areas and southern plains of the country.

## Ophthalmic nurses

An estimated 120 ophthalmic nurses currently serve in eye hospitals and eye departments, assisting ophthalmologists in operating theatres and pre- and post-operative care. Ophthalmic nurse training is not available in Nepal, and the hospitals recruit general nurses, who gain in-service exposure to become ophthalmic nurses.

## Other allied eye care personnel

The ophthalmic photographers do not have a formal training programme. Currently, about 15 OAs with an exposure and experience in clinical photography are present at major eye hospitals. Similarly, an estimated 20 orthoptists (ophthalmic assistants trained for a year) work at different tertiary eye hospitals. With the availability of hospital management training in the country, the number of eye hospitals run by trained managers or administrators is gradually increasing.

**Figure 3:**
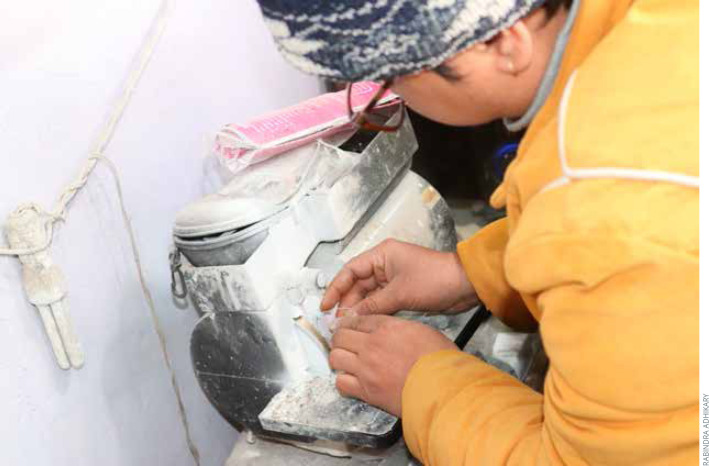
Most opticians in the rural areas rely upon manual edger for spectacle fitting.

In addition to these personnel, ‘eye workers’ provide supporting roles at hospitals and eye care centres across the country. The training for these workers is not standardised, and are based on the needs of the eye hospitals.

## Challenges

- Equitable distribution of the workforce is one of the major challenges faced by the allied eye care personnel. For example, the Karnali province, the least developed regions in Nepal has 17 OAs (1 OA per 90,000 people) compared to 210 (1 OA per 30,000 people) in Bagmati province. A similar pattern is likely for opticians and other allied eye care personnel.
- There are concerns about the retention of these professionals. Of the 1,025 registered OAs, only 625 are estimated to be active in the eye care sector. Factors such as poor job satisfaction, low salary and other incentives, lack of career growth, and an inappropriate match between the skills they have and those that the job demands may be responsible for demotivation and high attrition.
- Insufficient government involvement in eye care services has also led to fear about job security among all levels of the ophthalmic workforce.
- Training programmes for several of these personnel are not available in the country, and the programmes (e.g., optician and orthoptist) that are available are sporadic and lack standardisation.

## Opportunities

Despite these challenges, several opportunities exist to maximise the contribution of the allied ophthalmic personnel to eye care in the rural areas of Nepal. The National Ophthalmic Health Policy 2017 envisages integration of primary eye care into the existing primary health system, although this has not yet been implemented. The changing trend in eye diseases presents further opportunities for these personnel in primary eye care. Whereas cataract and refractive errors are major causes of vision impairment, the rising burden of diabetic retinopathy, glaucoma and other age-related eye diseases demands mobilisation of allied health personnel in awareness creation, early detection and primary prevention activities in an integrated health system. It is also encouraging to note that new training opportunities are being standardised for opticians.

## Conclusion

Allied ophthalmic personnel in Nepal have made a significant contribution to eye care services. However, their reach to the rural areas beyond district headquarters, is limited due to lack of integration into the existing primary health care system. Government job opportunities, standardised training, career opportunities, and incentives can help address the inequitable distribution and concentration of these personnel in urban regions. Further investigation is required to understand the effectiveness and impact of these professionals, as well as the factors associated with their recruitment and retention within the country's eye care sector.
